# Limited predictive value of pretreatment P-glycoprotein immunostaining for chemotherapy efficacy and survival in long-follow-up osteosarcoma patients

**DOI:** 10.1097/MD.0000000000043472

**Published:** 2025-08-08

**Authors:** Takashi Higuchi, Kentaro Igarashi, Shinji Miwa, Akihiko Takeuchi, Katsuhiro Hayashi, Satoru Demura, Norio Yamamoto, Hiroyuki Tsuchiya

**Affiliations:** a Department of Orthopaedic Surgery, Graduate School of Medical Sciences, Kanazawa University, Kanazawa, Isihikawa, Japan; b Kanazawa Red Cross Hospital, Kanazawa, Ishikawa, Japan.

**Keywords:** chemotherapy, immunohistochemistry, multidrug resistance protein, osteosarcoma, P-glycoprotein, survival

## Abstract

The clinical utility of P-glycoprotein (P-gp) in osteosarcoma remains controversial. This retrospective cohort study aimed to investigate the association between P-gp expression and clinicopathological factors, chemotherapy (CTx) response, and prognosis in osteosarcoma. Twenty-three osteosarcoma patients treated at our institution between 2007 and 2013 were included. P-gp expression rate was measured by immunohistochemical staining of biopsy specimens obtained at the time of diagnosis. CTx response was evaluated by image evaluation (Response Evaluation Criteria in Solid Tumors criteria) and pathological evaluation (Rosen and Huvos classification). The association between P-gp expression rate and clinicopathological factors, CTx response, disease-free survival (DFS), and overall survival (OS) was statistically analyzed. The P-gp expression rates ranged from 1.7% to 67.9% (mean 46.0%). No significant association was found between P-gp expression rate and surgical stage, CTx response, or clinical outcome. The receiver operating characteristic curve analysis revealed an optimal P-gp cutoff value of 47.0% for predicting poor CTx response, with a sensitivity of 73% and a specificity of 67%. When patients were divided into P-gp positive (n = 13) and negative (n = 10) groups based on the 47.0% cutoff value, no significant differences were observed between the 2 groups in terms of surgical stage, CTx response, OS, and DFS. Although the P-gp positive group showed a trend towards worse OS and DFS, no statistically significant differences were observed (OS: *P* = .73, DFS: *P* = .32). P-gp expression in osteosarcoma was not significantly associated with surgical stage, CTx response, or prognosis. These findings suggest that P-gp may not be a useful biomarker for CTx selection or prognosis prediction in osteosarcoma.

**Key points**

- No association between P-gp and osteosarcoma prognosis.
- P-gp is not a useful marker for osteosarcoma.
- The mechanism of multidrug resistance in osteosarcoma is complex.

## 1. Introduction

Osteosarcoma, the most common primary malignant bone tumor, predominantly affects adolescents and young adults.^[1,2]^ Despite advances in multimodal treatment, including surgery and chemotherapy (CTx), the prognosis for patients with metastatic or recurrent disease remains poor.^[3]^ Doxorubicin (DOX) is a cornerstone of first-line CTx for osteosarcoma, but the development of chemoresistance significantly limits its efficacy, leading to poor outcomes for many patients.^[4]^ Multidrug resistance (MDR) is a major obstacle to successful CTx in osteosarcoma. P-glycoprotein (P-gp), an adenosine triphosphate-dependent drug efflux pump, plays a critical role in mediating MDR by actively transporting chemotherapeutic agents, including DOX, out of cancer cells, thereby reducing their intracellular concentration and cytotoxic effect.^[5,6]^

While high P-gp expression has been reported to correlate with poor osteosarcoma prognosis, its clinical utility as a predictive biomarker is far from established.^[7–9]^ Studies yield conflicting results, with some demonstrating a positive association between P-gp levels and adverse outcomes, while others fail to find any significant correlation.^[7–9]^

Given the conflicting reports regarding the clinical utility of P-gp in osteosarcoma, we aimed to retrospectively investigate the association between P-gp expression, surgical stage, CTx response, and patient prognosis using immunohistochemistry (IHC) with a reliable P-gp antibody on biopsy specimens obtained at the time of diagnosis from patients treated at our institution. By examining these correlations, we determined whether P-gp serves as a clinically relevant biomarker for CTx selection and/or prognosis prediction in osteosarcoma.

## 2. Patients and methods

### 2.1. Patients

This retrospective study included 23 osteosarcoma patients who underwent CTx and surgery at our hospital (Kanazawa University Hospital, Kanazawa, Japan) between 2007 and 2013, for whom both biopsy pathological paraffin blocks and more than 10 years of long-term follow-up data were available (Table 1). There were 15 males and 8 females with a mean age of 20.6 years (range, 7–69) at the time of initial presentation. The primary sites were 12 femur, 8 tibia, 2 pelvis, and 1 clavicle. Histologically, the osteosarcomas were classified as osteoblastic (n = 16), chondroblastic (n = 4), telangiectatic (n = 2), and fibroblastic (n = 1). The surgical stages (Musculoskeletal Tumor Society staging system^[10]^) were 19 stage Ⅱb and 4 stage Ⅲ. All patients experienced neoadjuvant CTx. The mean number of preoperative CTx cycles was 6.1. The CTx protocols were based on standard doses of cisplatin (CDDP; 120 mg/m²), DOX (60 mg/m²), ifosfamide (9 g/m²), etoposide (180 mg/m²), methotrexate (10 g/m²), and vincristine (1.5 mg/m²). These doses were adjusted by the attending physician as appropriate for each patient’s weight, age, and clinical condition. The specific regimens included CDDP plus DOX, CDDP monotherapy, ifosfamide plus etoposide, and methotrexate plus vincristine. The mean total preoperative dose was 550.5 mg/m² for CDDP and 270.4 mg/m² for DOX. The mean treatment duration from the start of CTx to hospital discharge, including surgery and postoperative CTx, was 9.3 months. These patients were retrospectively evaluated for the P-gp positivity rate in the pretreatment biopsy specimens, the response to CTx (image evaluation and pathological evaluation), overall survival (OS), and disease-free survival (DFS). Image evaluation was performed using magnetic resonance imaging to assess tumor size changes before and after CTx based on Response Evaluation Criteria in Solid Tumors guideline (version 1.1).^[11]^ Pathological evaluation was performed according to the Rosen and Huvos classification.^[12]^ The present study was a retrospective cohort study approved by the Ethical Institutional Review Board of the Kanazawa University Hospital. Written informed consent was obtained from all study participants and/or their parents (in case of children).

**Table 1:** Patient characteristic (n = 23).

Patient characteristic	Value
Mean age (range), yr	20.6 (7–69)
Sex, n	
Male	15
Female	8
Osteosarcoma subtype	
Osteoblastic	16
Chondroblastic	4
Telangiectatic	2
Fibroblastic	1
Tumor location	
Femur	12
Tibia	8
Pelvis	2
Clavicle	1
Chemotherapy (mean cycles), n	
Preoperative	23
DOX + CDDP (5.1)	11
DOX + CDDP (3.2), IFO + VP-16 (3.4)	9
DOX + CDDP (7), CDDP (1)	1
DOX + CDDP (8), MTX + VCR (1)	1
DOX + CDDP (6), CDDP (1), MTX + VCR (1)	1
Postoperative	23
Surgery, n	23
Mean treatment duration, months	9.3
Mean follow-up period, months	
All patients	122.4
Survived patients	156.6

CDDP = cisplatin, DOX = doxorubicin, IFO = ifosfamide, MTX = methotrexate, VCR = vincristine, VP-16 = etoposide.

### 2.2. Histological analysis

**Figure 1.**
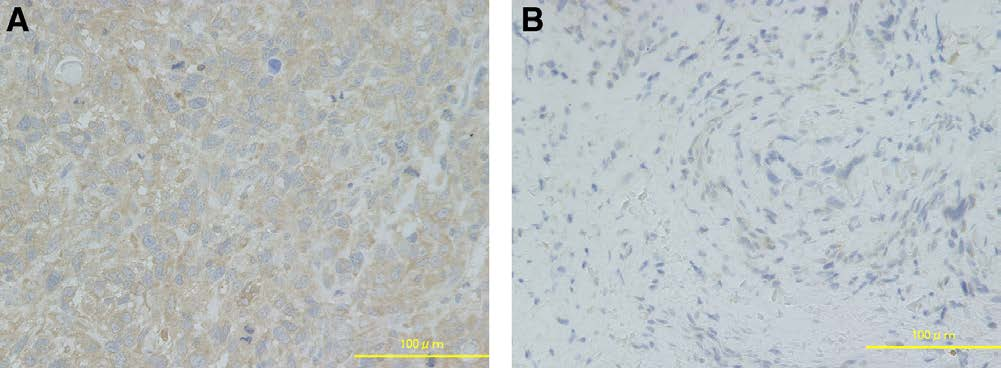
Representative immunohistochemical staining for P-gp in osteosarcoma biopsy specimens. (A) Immunohistochemical staining for P-gp in a specimen from a 12-year-old female with distal femur osteosarcoma, demonstrating positive P-gp expression. (B) Immunohistochemical staining for P-gp in a specimen from a 16-year-old male with distal femur osteosarcoma, demonstrating negative P-gp expression. Scare bars indicate 100 µm. P-gp = P-glycoprotein.

Paraffin-embedded tissue blocks of the diagnostic biopsy specimens were retrieved from our institutional pathology archives. These blocks were sectioned, deparaffinized, and rehydrated in xylene and ethanol, then IHC stained using an antibody against P-gp (PGP Polyclonal Antibody, Proteintech Japan Inc., Tokyo, Japan) in combination with diamino-benzidine (DAB, Dako Japan Inc., Kyoto, Japan) staining, and hematoxylin counterstaining (Fig. 1).

### 2.3. P-gp expression rate and cutoff value

IHC staining for P-gp was performed on biopsy specimens from all patients. Since P-gp is predominantly expressed in the cytoplasm of cancer cells, the P-gp expression rate was evaluated by calculating the ratio of the stained area to the total cell area. ImageJ software was used for this quantitative analysis of stained and total cell areas. The P-gp expression rate was determined by evaluating 6 randomly selected fields of view and calculating the average expression rate for each patient. A receiver operating characteristic (ROC) curve was generated using the P-gp expression rate for each patient and the presence of a poor CTx response. Patients with stable disease or progressive disease were defined as having a poor response to CTx. The P-gp cutoff value that maximized sensitivity and specificity was determined from the ROC curve.

### 2.4. Statistical analysis

Statistical analyses were performed using EZR (Saitama Medical Center, Jichi Medical University, Saitama, Japan), which is a graphical user interface for R (The R Foundation for Statistical Computing, Vienna, Austria) and has medical statistical functions including survival analyses and competing risk analyses based on R (version 2.3-0).^[13]^ Differences between 2 groups were analyzed using Student *t* test. Differences among 3 or more groups were analyzed using one-way analysis of variance. Fisher exact test was used to compare proportions between groups. Survival differences were analyzed using the Log-rank test. A *P*-value of <.05 was considered statistically significant.

## 3. Results

### 3.1. P-gp positivity and surgical stage, CTx response, and clinical outcome

**Figure 2.**
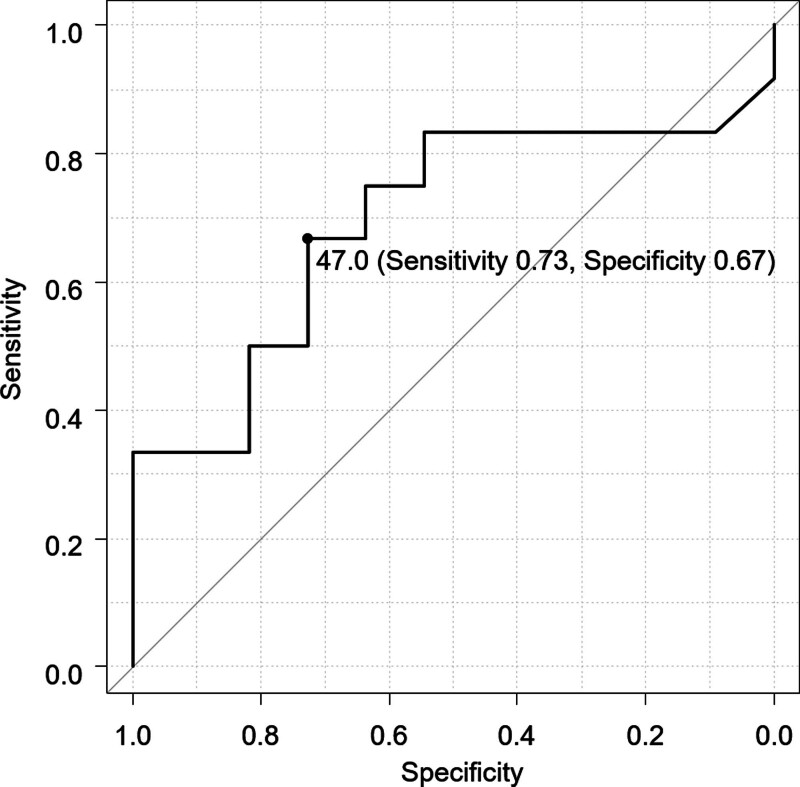
Receiver operating characteristic (ROC) curve analysis for P-gp expression rate in predicting poor chemotherapy response. ROC curve analysis revealed a P-gp cutoff value of 47.0% that maximized sensitivity and specificity for predicting poor chemotherapy response. P-gp = P-glycoprotein.

The P-gp expression rates ranged from 1.7% to 67.9%, with a mean of 46.0%. No significant association was found between P-gp expression rate and surgical stage (*P* = .15), CTx response, as assessed by both image evaluation (*P* = .67) and pathological evaluation (*P* = .42), and clinical outcome (*P* = .72) (Table 2). The ROC curve analysis revealed a P-gp cutoff value of 47.0% that maximized sensitivity and specificity for predicting poor CTx response (Fig. 2). The sensitivity and specificity were 0.73% and 0.67%, respectively (Fig. 2).

**Table 2:** P-gp expression rates among surgical stage, chemotherapy response, and clinical outcome.

(n)	P-gp expression rate	*P*-value
Surgical stage		
Ⅱ B (19)	41.0	.15
Ⅲ B (4)	22.6	
MRI based CTx response		
PD (3)	40.4	.67
SD (5)	48.9	
PR (8)	32.9	
CR (7)	34.4	
Pathologic CTx response		
Ⅰ (4)	36.1	.42
Ⅱ (10)	34.4	
Ⅲ (3)	25.3	
Ⅳ (6)	50.8	
Clinical outcome		
DOD (6)	32.6	.72
AWD (3)	45.6	
NED (1)	17.8	
CDF (13)	39.9	

*P*-values in each section were analyzed with Student *t* test or one-way analysis of variance.

AWD = alive with disease, CDF = continuous disease free, CR = complete response, CTx = chemotherapy, DOD = dead of disease, MRI = magnetic resonance imaging, NED = no evidence of disease, PD = progressive disease, P-gp = P-glycoprotein, PR = partial response, SD = stable disease.

When patients were divided into P-gp positive (n = 13) and negative groups (n = 10) based on this 47% cutoff value, no significant differences were observed between the 2 groups in terms of surgical stage (*P* = .60), CTx response assessed by image evaluation (*P* = .64) and pathological evaluation (*P* = .39), and clinical outcome (*P* = .91) (Table 3).

**Table 3:** Number of patients in the P-gp positive and negative groups.

P-gp (expression rate)	Positive (>47%)	Negative (<47%)	*P*-value
Whole	10	13	
Surgical stage			
Ⅱ B	9	10	.60
Ⅲ B	1	3	
MRI based CTx response			
PD	2	1	.64
SD	3	2	
PR	3	5	
CR	2	5	
Pathologic CTx response			
Ⅰ	2	2	.39
Ⅱ	3	6	
Ⅲ	0	3	
Ⅳ	4	2	
Clinical outcome			
DOD	3	3	.91
AWD	2	1	
NED	0	1	
CDF	5	8	

*P*-values in each section were analyzed with Fisher exact test.

AWD = alive with disease, CDF = continuous disease free, CR = complete response, CTx = chemotherapy, DOD = dead of disease, MRI = magnetic resonance imaging, NED = no evidence of disease, PD = progressive disease, P-gp = P-glycoprotein, PR = partial response, SD = stable disease.

### 3.2. P-gp positivity and survival

**Figure 3.**
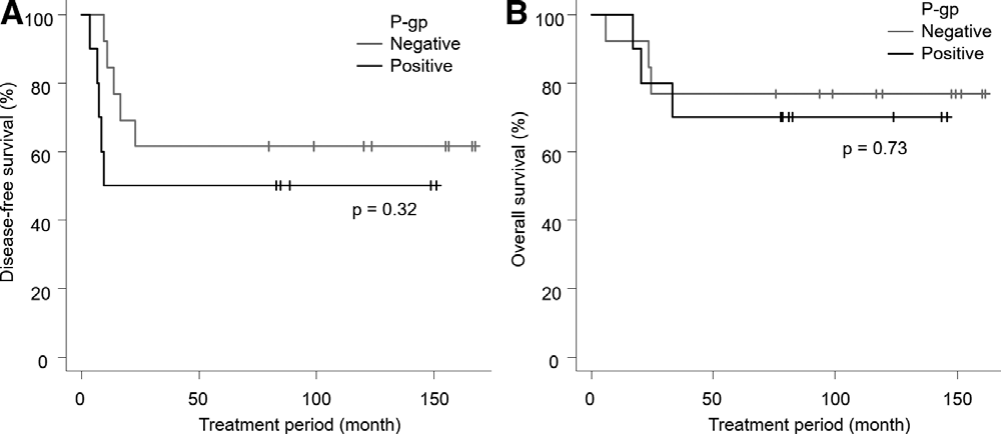
Survival curves in P-gp positive and negative groups. (A) Survival curves of disease-free survival (DFS) in patients with P-gp positive and negative osteosarcoma, compared using the Log-rank test. (B) Survival curves of overall survival (OS) in patients with P-gp positive and negative osteosarcoma, compared using the Log-rank test. P-gp = P-glycoprotein.

The mean follow-up period was 122.4 months for all patients, and 156.6 months for survived patients. The DFS rate at 5 years were 50.0% in positive group and 61.5% in negative group (Fig. 3A). Among the 23 patients, 10 experienced recurrence or metastasis during the observation period, resulting in a relapse rate of 43.4%. The OS rates at 5 years were 70.0% in P-gp positive group and 76.9% in negative group (Fig. 3B). No significant association was found between P-gp expression rate and DFS or OS. Although the P-gp positive group tended to be worse DFS and OS, no statistically significant differences were observed in either DFS (*P* = .32) or OS (*P* = .73) between the 2 groups (Fig. 3).

## 4. Discussion

The ability to reliably assess drug resistance before treatment is crucial to avoid potential drug toxicity from ineffective agents and to improve patient prognosis. Doxorubicin, an anthracycline, remains a key drug of osteosarcoma treatment, but its efficacy is often compromised by chemoresistance.^[14,15]^ Chemoresistance to doxorubicin, particularly MDR mediated by P-gp, which was the target of this study, poses a significant clinical challenge.^[16]^ Furthermore, the cumulative dose-dependent cardiotoxicity associated with doxorubicin requires a delicate balance between therapeutic efficacy and patient safety in osteosarcoma patients who require long-term treatment regimens.^[17]^ Therefore, individualized strategies, such as knowing doxorubicin resistance in advance and limiting dosing to doxorubicin-resistant patients, are desirable.

The clinical utility of pretreatment P-gp detection has been investigated in various malignancies, but its value remains controversial. While some studies have reported a significant association between high P-gp expression and poor prognosis or chemoresistance such as childhood soft-tissue sarcoma and breast cancer, others have failed to demonstrate such a correlation such as colorectal cancer and ovarian carcinoma.^[18–21]^ This discrepancy may be attributed to several factors, including differences in tumor types, P-gp detection methods, and the complex interplay of other resistance mechanisms.

In osteosarcoma, the role of P-gp as a predictive biomarker is more challenging to define, as evidenced by the conflicting results in previous studies. While some studies have reported a significant association between high P-gp expression and poor prognosis, indicating that P-gp overexpression may confer a survival disadvantage by promoting drug resistance and tumor progression, others, including the present study, have failed to demonstrate such a correlation, suggesting that P-gp expression may not be an independent prognostic factor in osteosarcoma.^[7–9,22,23]^

This discrepancy in findings may be attributed to several factors, including differences in patient populations, treatment protocols, and P-gp detection methods, particularly variations in immunohistochemical antibodies and scoring methodologies employed to assess P-gp expression.^[22]^ Furthermore, the impact of P-gp on prognosis may be influenced by the specific subtype of osteosarcoma, the presence of other resistance mechanisms, and the tumor microenvironment.^[24–26]^

Using tissue microarray to evaluate P-gp expression in osteosarcoma, which minimizes variability caused by tissue fixation, antigen retrieval, and staining methods in IHC, was reported to reveal a correlation between P-gp expression and both poor response to CTx and metastasis development, but not survival.^[22]^

The heterogeneity of osteosarcoma, both inter- and intra-tumorally, may contribute to the variability in P-gp expression and its impact on treatment response. The complex interplay of multiple resistance mechanisms, including alterations in drug targets, enhanced deoxyribonucleic acid repair, and activation of survival signaling pathways, may overshadow the isolated effect of P-gp.^[5,27]^

In addition to P-gp, other adenosine triphosphate-binding cassette transporters, such as multidrug resistance-associated protein 1 and breast cancer resistance protein, have been implicated in MDR in osteosarcoma.^[28]^ These transporters may contribute to drug efflux and resistance through overlapping or distinct substrate specificities.^[29]^

The tumor microenvironment, including hypoxia, inflammation, and the presence of cancer stem cells, may influence drug resistance in osteosarcoma.^[26]^ These factors may modulate P-gp expression and function, as well as other resistance mechanisms, through complex signaling pathways.^[9]^

The lack of a significant correlation between P-gp expression and clinical outcomes in our study may also be attributed to the relatively small sample size and the retrospective nature of the analysis. A larger, prospective study with standardized treatment protocols and a more diverse patient population is warranted to validate our findings and to explore the potential of P-gp as a predictive biomarker in osteosarcoma.

Therefore, the interpretation of our findings should be considered within the context of these conflicting reports and the complex interplay of factors that influence osteosarcoma prognosis. Future studies should aim to address these discrepancies by utilizing standardized methodologies, larger patient cohorts from multi-center, and comprehensive analyses of other prognostic factors.

In conclusion, our study did not demonstrate a significant association between P-gp expression and clinical outcomes, CTx response, and prognosis in osteosarcoma, suggesting that P-gp may not be a useful biomarker for CTx selection or prognosis prediction in osteosarcoma. However, the role of P-gp in osteosarcoma chemoresistance remains complex and warrants further investigation. Future studies should focus on larger, prospective cohorts, standardized treatment protocols, and the integration of multi-omics approaches to elucidate the intricate mechanisms of drug resistance and to identify clinically relevant biomarkers for personalized osteosarcoma therapy.

## Author contributions

**Conceptualization:** Takashi Higuchi.

**Formal analysis:** Takashi Higuchi.

**Funding acquisition:** Takashi Higuchi.

**Investigation:** Kentaro Igarashi, Shinji Miwa.

**Supervision:** Satoru Demura, Hiroyuki Tsuchiya.

**Writing – original draft:** Takashi Higuchi.

**Writing – review & editing:** Akihiko Takeuchi, Katsuhiro Hayashi, Norio Yamamoto.
